# Influence of Summer Drought on Post-Drought Resprouting and Leaf Senescence in *Prunus spinosa* L. Growing in a Common Garden

**DOI:** 10.3390/plants14071132

**Published:** 2025-04-05

**Authors:** Kristine Vander Mijnsbrugge, Stefaan Moreels, Sharon Moreels, Damien Buisset, Karen Vancampenhout, Eduardo Notivol Paino

**Affiliations:** 1 Department of Forest Ecology and Management, Research Institute for Nature and Forest, 9500 Geraardsbergen, Belgium; stefaan.moreels@inbo.be (S.M.); sharon.moreels@inbo.be (S.M.); damien.buisset@ulb.be (D.B.); 2 Department of Earth and Environmental Sciences, KU Leuven Campus Geel, Kleinhoefstraat 4, 2440 Geel, Belgium; karen.vancampenhout@kuleuven.be; 3 Department for Environment, Agricultural and Forest Systems, Agri-Food Research and Technology Centre of Aragon (CITA), 50059 Zaragoza, Spain; enotivol@cita-aragon.es

**Keywords:** water limitation, dry-out experiment, provenance trial, black thorn, leaf discoloration, recovery, phenology, bud burst

## Abstract

Understanding how woody plants cope with severe water shortages is critical, especially for regions where droughts are becoming more frequent and intense. We studied the effects of drought intensity, focusing on post-drought resprouting, autumn leaf senescence and the subsequent spring bud burst. Furthermore, we aimed to study population differentiation in the drought and post-drought responses. We performed a summer dry-out experiment in a common garden of potted *Prunus spinosa* L. (Rosaceae) saplings. We analysed responses across different visual stress symptom categories and examined differentiation between provenances from a local origin (Western Europe, Belgium), a lower latitude (Spain) and a higher latitude (Sweden). The chance of post-drought resprouting was greater for the more severely affected plants than for the less severely affected ones, and it occurred earlier. The plants that displayed wilting of the leaves during the drought had a leaf senescence 2.7 days earlier than the controls, whereas that of plants with 25 to 75% and more than 75% of desiccated leaves was 7 and 15 days later, respectively. During the drought, the local provenance was the first to develop visual symptoms compared to the other two provenances. However, among plants that exhibited no or only mild symptoms, this provenance also had a higher likelihood of post-drought resprouting. Among the control plants, the higher-latitude provenance displayed leaf senescence earlier, while the lower-latitude provenance senesced later compared to the local provenance. However, these differences in the timing of leaf senescence among the three provenances disappeared in treated plants with more than 25% of desiccated leaves due to the drought. Whereas leaf senescence could be earlier or later depending on the developed drought symptoms, the timing of bud burst was only delayed. Results indicate that resprouting and timing of leaf senescence are responsive to the severity of the experienced drought in a provenance-dependent way.

## 1. Introduction

Richly structured forest edges can serve as biodiversity hotspots for both fauna and flora [1,2]. The rise in extreme weather events like droughts and heatwaves due to climate change may lead to higher tree and shrub mortality rates, potentially causing a net release of CO_2_ in the atmosphere. Among these extreme events, drought and its related disturbances have the greatest global impact on forests and wooded lands [3,4]. Drought is expected to increase in frequency and severity in many regions in the future resulting from global climate change [5,6]. Trees and shrubs may not be able to adapt in time to increases in aridity through evolutionary mechanisms due to their long reproductive life cycles and limited capacity to move away from stressful environments. The imminent threat of extended and more severe droughts underscores the need to study drought effects on woody plants [4,6].

During water limitation, plants eventually undergo desiccation, leading to a decrease in cell turgor pressure and causing the stomatal pores on leaf surfaces to close. This closure significantly slows down the dehydration process [7]. Wilting of the leaves and the loss of stem conductivity occur when drought stress becomes severe [8,9]. The light-harvesting capacity of leaf photosynthesis is damaged only after a significant loss of hydraulic function under prolonged dehydration [10]. During extreme drought, leaf shedding is an adaptive strategy to enhance survival chances [11,12]. Typically, leaf shedding occurs after stomatal closure [13], with xylem embolism in the leaves being a primary cause of leaf mortality during drought [14,15,16]. The shedding reduces the evaporative leaf surface area, helping woody perennials delay cavitation in stem conductive tissues [17]. However, shedding leaves without fully resorbing the nutrients results in net nutrient loss, which can influence the functioning of the tree in the long run [17].

While much focus has been placed on understanding the physiological aspects contributing to drought-induced tree mortality, it is likewise crucial to comprehend the mechanisms that are involved in drought recovery. Resilience to dehydration can be evaluated by examining both the impact of the drought and the rate of post-drought recovery [18]. If cavitation of the conducting tissues is minimal or non-existent, recovery after re-watering is swift, with stomata reopening, allowing new carbon to be assimilated [19]. However, if cavitation thresholds are surpassed, recovery of photosynthesis is slower [19]. Many plant species can resprout vegetatively after substantial biomass loss caused by environmental stress, including drought [20]. Quite evidently, plants require adequate storage reserves to resprout after the loss of shoots and foliage. These reserves include non-structural carbohydrates stored in roots and stems [20,21].

Senescence is the final stage in the life cycle of leaves, and in deciduous woody species, it signals the transition from the active to the dormant stage [12,22]. It marks a shift from nutrient assimilation to nutrient remobilization, which is vital for plant fitness [12]. This involves a gradual and coordinated disassembly of macromolecules, leading to nutrient accumulation, which is then mobilised away from the senescing leaves [23]. When leaves become older, they become more permissive to the induction of senescence and, at the same time, remain competent for perceiving senescence-delaying or -reverting signals [24]. Leaf senescence is governed by complex genetic programs, finely regulated at multiple levels [23,25] , and is influenced by various environmental stresses [12,26]. The literature provides mixed reports on whether drought stress leads to earlier, later, or unchanged timings of autumn leaf senescence, which complicates our understanding of its effects. Drought stress can advance leaf senescence [22,27]. This is supported by observations of early leaf abscission due to hydraulic failure in response to drought [12]. Other studies report that drought stress can delay autumn leaf senescence [28,29]. No difference in the timing of leaf senescence upon drought stress has been reported [26].

The primary objective of this study is to improve our understanding of how woody plants respond to summer droughts. This knowledge will contribute to more accurate predictions of ecosystem responses to future climate challenges and support conservation strategies aimed at preserving biodiversity and ecosystem services in increasingly drought-prone environments. In addition, within species, responses to drought are not necessarily similar for different populations originating from diverse geographic origins, and a better knowledge of population differentiation in drought responses can help decisions on assisted migration as an anticipation of climate change. In this study, we carried out a controlled experiment in greenhouse conditions to assess the effects of drought on the shrub *Prunus spinosa* L. (Rosaceae). The experiment was performed with three provenances in a common garden setting. Potted saplings were subjected to water deprivation during the summer of 2021, followed by rewatering. We hypothesised that the responses to the water withholding would be affected by (i) the severity of the drought and (ii) the provenance of the saplings. Our objective was to gain more understanding of the post-drought recovery process, with a specific focus on the ability for post-drought resprouting and on the timing of leaf senescence. The common garden allowed the assessment of the variability in responses among the different provenances. We were able to relate visual symptoms of drought stress with post-drought responses. Most strikingly, leaf senescence was advanced or delayed depending on the severity of the drought stress. We also observed population differentiation in the drought responses.

## 2. Results

### 2.1. Initial Traits

**Figure 1:**
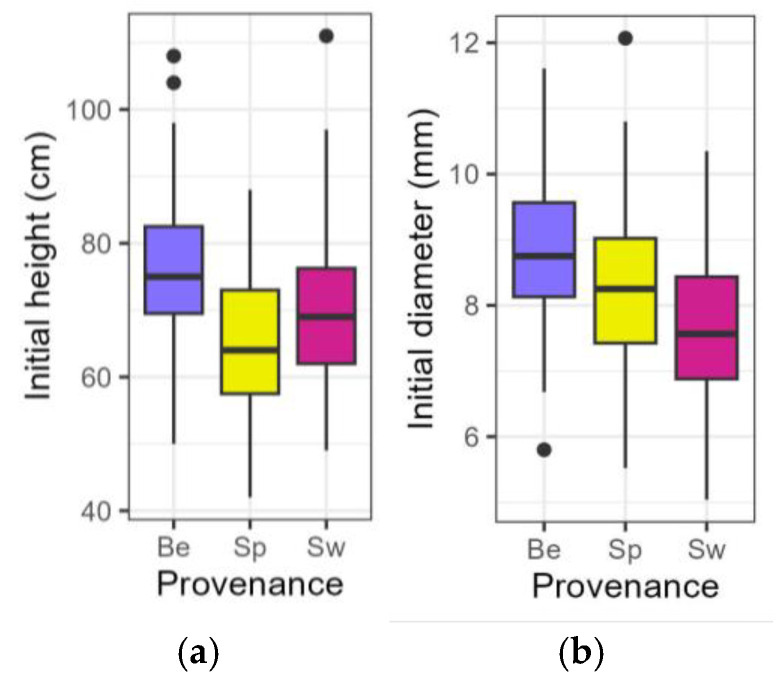
Boxplots presenting the initial height (**a**) and diameter (**b**) of the saplings at the start of the treatment. Be: Belgian, Sp: Spanish-Pyrenean, Sw: Swedish.

At the start of the treatment, plants from the Belgian provenance were the highest and also displayed the largest diameter compared to the other two provenances (Table 1, Figure 1a,b). Also, plants from the Belgian provenance were characterised by relatively larger leaves. Long shoot leaves from the Belgian provenance were larger (length and widest width) than from the Spanish-Pyrenean, whereas the short shoot leaves from the Belgian provenance were longer than the Spanish-Pyrenean and wider than the Swedish provenance (Figure S1, Table S1). Stomatal density in the Belgian provenance did not differ from the other two provenances, nor did the stomatal length (Figure S2, Table S2).

### 2.2. Development of Visual Drought Symptoms

**Figure 2:**
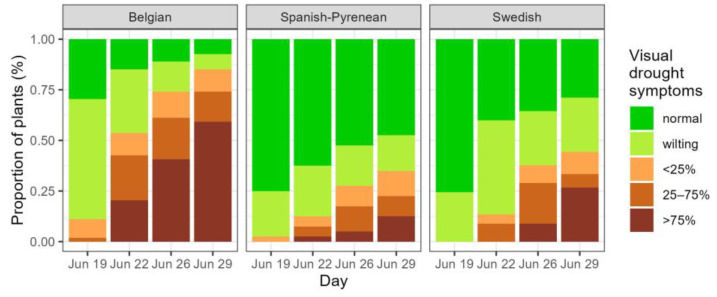
Development of visual drought symptoms during the drought period in the three provenances, with *p* values for the fixed effects in the mixed model. Normal: no visual drought symptoms; wilting: leaf wilting, <25%, 25–75% and >75% of desiccated leaves.

In the drought-treated group of plants, visual drought symptoms started to develop and were scored several times. Plants from the Spanish-Pyrenean and Swedish provenances displayed visual symptoms significantly later than the Belgian provenance (significant provenances in Table 2, Figure 2).

### 2.3. Post-Drought Resprouting

**Figure 3:**
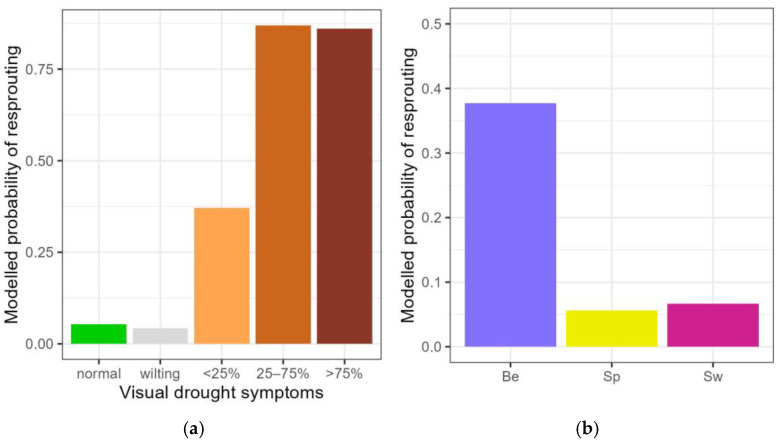
Modelled probability of post-drought resprouting among the different categories of visual drought symptoms (**a**) and among the three provenances for the pooled visual drought categories normal, wilting leaves and <25% desiccated leaves (**b**). Normal: no visual drought symptoms; wilting: leaf wilting, <25%, 25–75% and >75% of desiccated leaves. Be: Belgian, Sp: Spanish-Pyrenean, Sw: Swedish. Categories not significantly differing from the standard (normal) are displayed in grey.

Some of the droughted plants resprouted after the rewatering, whereas others did not. First, a model was run to examine the resprouting ability in the different levels of visual drought symptoms for all the droughted plants. Plants with desiccated leaves at the end of the drought period (<25%, 25–75% and >75% of desiccated leaves) displayed a significantly higher chance for post-drought resprouting than the plants without visual drought symptoms (significant drought categories <25%, 25–75% and >75% of desiccated leaves in Table 3). The modelled chance of resprouting for the plants in the category < 25% of desiccated leaves was in between the chances of normal plants and plants with more than 25% of desiccated leaves (Figure 3a).

Secondly, we looked at putative differences in the resprouting response among the plants of the three provenances. Modelling the plants with visual drought symptoms up to 25% of desiccated leaves (normal, wilting and <25% desiccated leaves) revealed that the chance to resprout was lower for the Spanish-Pyrenean and the Swedish provenance compared to those of Belgian provenance (significant provenances in Table 3, Figure 3b). Among the plants that displayed severe drought symptoms (25–75% and >75% of desiccated leaves), there was no longer a significant difference among the three provenances in the modelled chance for post-drought resprouting (no significant values for the provenances in Table 3).

**Figure 4:**
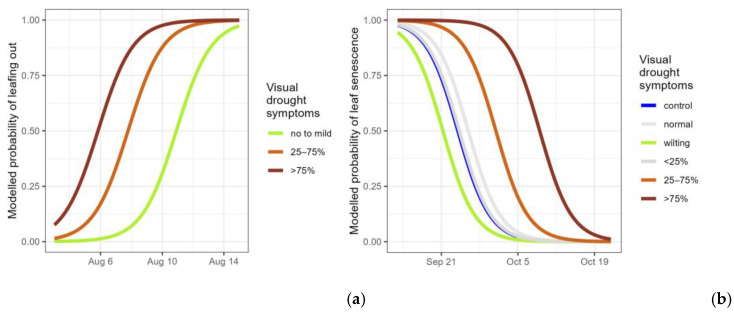
Modelled probability of the timing of resprouting among the resprouting saplings (having new leaves emerging but not yet protruding from the bud) (**a**) and modelled probability of the timing of leaf senescence for all controls and droughted plants (still having green leaves) (**b**), according to the visual drought symptom categories. For the resprouting (**a**), no to mild: pooling of no visual drought symptoms, wilting leaves and <25% of desiccated leaves; 25–75% and >75% of desiccated leaves. For leaf senescence (**b**), normal: no visual drought symptoms; wilting: leaf wilting, <25%, 25–75% and >75% of desiccated leaves. Categories not significantly differing from the standard (control) are displayed in grey.

For the droughted plants that resprouted after rewatering (n = 64), the timing of resprouting was modelled. The first model focused on the different categories of visual drought symptoms. Because of the low number of plants in the categories with no to mild drought symptoms (score 1, 2 and 3), these were pooled, resulting in a visual drought symptoms variable with three levels: no to mild symptoms (n = 9), 25–75% desiccated leaves (n = 13) and more than 75% (n = 42). The last two categories resprouted significantly earlier (significant categories for visual drought symptoms in Table 4, Figure 4a).

When looking at differentiation among the provenances for the timing of post-drought resprouting (Belgian n = 38, Spanish-Pyrenean n = 11, Swedish n = 15) we could not take into account the different visual drought symptom categories in the model because of the low number of plants in each subgroup. No provenance differentiation was present (no significant provenances in Table 4).

### 2.4. Autumn Leaf Senescence

The timing of autumn leaf senescence was modelled for all the plants in the experiment. The first focus was to look for differentiation in responses among the control plants and the different categories of visual drought symptoms. The timing of leaf senescence was 2.7 days earlier for the droughted plants that displayed wilted leaves (score 2) in comparison to the control plants (significant categories of visual drought symptoms in Table 5, Figure 4b). Droughted plants with less than 25% of desiccated leaves (score 3) displayed no difference in timing compared to the control plants, whereas the timing was 7 and 15 days later for plants with more than 25% of desiccated leaves (scores 4 and 5, respectively) (significant categories of visual drought symptoms in Table 5, Figure 4b). Finally, droughted plants without visual stress symptoms (score 1) did not differ from the control plants.

**Figure 5:**
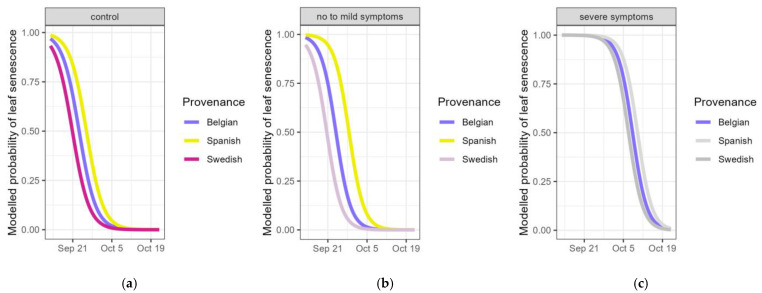
Modelled probability of the timing of leaf senescence (still having green leaves) according to the provenance for controls (**a**), pooled categories of visual drought symptoms normal, wilting and <25% desiccated leaves (**b**) and pooled categories of visual drought symptoms 25–75% and >75% desiccated leaves (**c**). Provenances not significantly differing from the standard (Belgian) are displayed in grey.

Secondly, the timing of autumn leaf senescence was compared between the plants of the different provenances. For the control plants, the timing of leaf senescence in the Spanish-Pyrenean provenance was 2.6 days later than in the Belgian provenance, and it was 2.4 days earlier in the Swedish provenance (significant provenances in Table 5, Figure 5a). In the pooled categories of no to mild visual drought symptoms (scores 1, 2 and 3) in the drought-treated plants, the timing for the Spanish-Pyrenean provenance was 4.7 days later than the Belgian provenance and there was a tendency (*p*-value = 0.051 in Table 5) for the Swedish provenance to be earlier (3.3 modelled days) (significant provenances in Table 5, Figure 5b). For the droughted plants that displayed severe symptoms (more than 25% of the leaves desiccated due to the drought: pooled scores 4 and 5), the timing of leaf senescence no longer differed among the three provenances (no significant provenances in Table 5, Figure 5c). Based on the models, the time span between no to mild (pooled scores 1, 2 and 3) and severe visual drought symptoms (pooled scores 4 and 5) was 16.2 days for the Swedish provenance, 14.4 days for the Belgian and 11.5 days for the Spanish-Pyrenean provenance.

**Figure 6:**
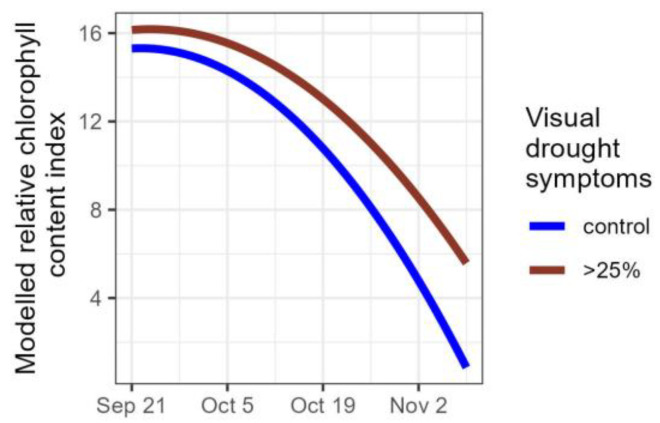
Modelled relative chlorophyll content index for control plants (n = 27) and for plants severely affected by the drought, i.e., that lost more than a quarter of their foliage (n = 27).

The relative chlorophyll content in a subset of control plants was compared with a subset of severely affected plants (plants that lost more than a quarter of their foliage due to the drought). In both groups the relative chlorophyll content decreased, but it decreased at a faster rate in the control group than in the group of severely affected plants (significant interaction term between the time variable and the drought variable in Table 6, Figure 6), corroborating the results of the leaf senescence scores, with plants that had more than 25% of their leaves desiccated due to the drought, senescing significantly later than the control plants.

### 2.5. Timing of Bud Burst

**Figure 7:**
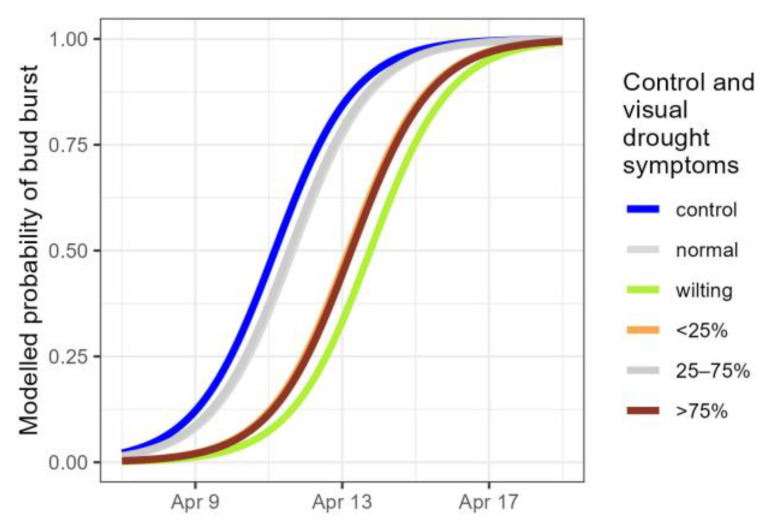
Modelled probability of the timing of bud burst for controls and droughted plants, according to the visual drought symptom categories. Normal: no visual drought symptoms; wilting: leaf wilting, <25%, 25–75% and >75% of desiccated leaves. Categories not significantly differing from the standard (control) are displayed in grey.

In the year following the treatment, the plants that displayed wilting and desiccation in less than 25% of the leaves, as well as the plants with more severe visual drought symptoms (>75% desiccated leaves) were characterised by a delayed bud burst (significant categories of visual drought symptoms in Table 7, Figure 7). The group of plants that displayed an earlier leaf senescence (wilting of the leaves during the drought), the intermediate group that did not deviate in leaf senescence from the controls (<25% desiccated leaves), and one of the groups that showed a later senescence (>75% desiccated leaves), were all characterised by a significantly later bud burst (Table 8).

For the timing of bud burst among the plants from the different provenances, the dataset was split into the same three groups as for the timing of leaf senescence. The Swedish provenance burst its buds later than the Belgian provenance in all three datasets (significant provenances in Table 7, Figure S3).

## 3. Discussion

In our common garden experiment, we examined the response of *P. spinosa* to summer drought. Specifically, we analysed post-drought resprouting, the timing of leaf senescence, and the timing of bud burst in the following year. For each aspect, we first discuss the observed mechanisms and then examine population differentiation.

### 3.1. Post-Drought Resprouting

Woody plants can experience significant damage to their foliage during severe drought conditions. However, many species have the ability to recover and produce new foliage after the drought has ended. Our study species, *P. spinosa*, was already shown to hold the capacity for resprouting after severe loss of above-ground biomass by burning or cutting [30]. In woody plants in general, this resprouting response has been defined in two ways: binary or continuous [31,32]. In a binary framework, a plant either dies or resprouts and survives. A continuous framework defines the resprouting response as a spectrum, ranging from weak to strong reactions to the disturbance. Our experiment corroborates the continuous framework with a higher chance of post-drought resprouting for the more severely affected plants. When stress severity increases, the cost of carbon needed to reestablish functionality upon stress release also rises [33]. This seems to contradict the earlier onset of resprouting in the most severely affected plants in our experiment. Most probably, this quick reaction underpins the urge with which these most severely affected plants need the newly formed foliage to maximise their chances on survival. This may come at a higher risk of finally dying off when a new drought or other types of stress, such as an early autumn frost, may occur.

Concerning population differentiation in the common garden, we observed that plants from the local provenance were the first to exhibit visual drought symptoms during the water withholding period, possibly partly governed by larger leaves and, thus, likely a larger transpiration area. At the interspecies level, large-leaved species occupy, in general, rather wet and hot environments, whereas small leaves are found more at higher latitudes and higher elevations [34]. This general rule may hold true intra-specifically [35,36], explaining the smaller leaves in non-local provenances, which could have contributed to the later onset of drought symptoms. Interestingly, the local provenance also exhibited a higher chance of post-drought resprouting when comparing groups with equal visual drought symptoms, suggesting that this provenance is not only characterised by a higher vulnerability to sudden and severe drought but also by a stronger resprouting response to recover from it.

### 3.2. Advancement or Delay of Autumn Leaf Senescence

In our common garden experiment, we observed that the drought-treated plants exhibited a timing of leaf senescence that ranged from earlier to later than the controls. A similar “two strategies” response was already described for another common shrub, *Cornus sanguinea* L. [37]

An earlier leaf senescence is a well-known response to drought [12,27,38]. Although global warming delays the autumn date of foliar senescence, warming-related drought may counteract this by causing earlier foliar senescence due to water limitation [39]. Post-drought advancement of leaf senescence allows the plants to avoid nutrient loss due to limited resorption from desiccating leaves during a potential future drought [17]. Interesting to note is that the earlier senescence in our study concurred with the wilting of the leaves during the preceding drought. As leaf desiccation is related to hydraulic failure in the vascular tissue of the leaf [13], which may imply that turgor loss but not yet harm to the conductive tissues of the leaves produced the signals for the observed advancement. Abscisic acid is the well-studied signalling molecule that affects stomatal closure during drought [40] but does not directly induce earlier autumn leaf senescence [41]. Possibly, the accumulation of Reactive Oxygen Species, also an early response upon drought that leads to the closure of stomata [42], may have induced the advancement of autumn leaf senescence [43].

After severe drought stress, the demand for carbon to restore hydraulic conductivity increases [33], likely compelling plants to delay leaf senescence. This strategy, however, comes with increased risks, including higher mortality if new drought events occur and greater susceptibility to injury from early autumn frosts [22]. Since mortality is associated with critical hydraulic failure [44], the risky delayed leaf senescence might represent a last effort to survive. A delay in leaf senescence following drought has been previously reported in *Fagus sylvatica* L. [45] and *Quercus petraea* (Matt.) Liebl [28]. In drought experiments on *F. sylvatica*, increased photosynthesis was detected after drought release [45,46]. In our experiment, the later senescence in the group of plants with more than a quarter of their foliage desiccated by the drought was corroborated by relative chlorophyll content measurements.

Similar to *C. sanguinea* [37], a group of droughted plants with intermediate visual drought symptoms (desiccated leaves up to 25%) did not deviate in the timing of leaf senescence with the control group. It can be hypothesised that the two contrasting responses, earlier and later leaf senescence, cancel each other out.

Interestingly, when looking at population differentiation for the timing of leaf senescence in the common garden, we observed that the differentiation among the three provenances differed among the treatment groups. Whereas for the control plants, leaf senescence was later in the Spanish-Pyrenean provenance and earlier in the Swedish provenance, compared to the local Belgian, these differences disappeared among the plants heavily affected by the drought that senesced later. The synchronisation of this phenophase among the provenances in the severely affected plants may suggest that the signals for the onset and progression of leaf senescence are both genetically and environmentally controlled, showing a classic example of phenotypic plasticity with an environmental threshold that exceeds and dilutes the genetic pattern [47]. The later the leaf senescence occurs, the more the differentiation among the provenances may fade due to altered environmental conditions, such as photoperiod and temperature [47]. From repeated provenance trials at different geographic locations, it is known that the phenological behaviour of provenances is not only determined by genetic factors but also by the local conditions of the provenance trial [48,49].

### 3.3. Bud Burst in the Year Following the Drought Treatment

Another interesting effect was observed in the phenological trait bud burst in the year following the treatments. A delay in bud burst timing, or no difference, was noticed when comparing the drought-treated groups with the controls, but no advancement. It can be hypothesised that the levels of non-structural carbohydrates contribute to these results. Post-drought repair of embolism in the conductive tissue positively correlated to stem non-structural carbohydrate depletion due to the drought, with the magnitude of hydraulic recovery positively correlating to the consumption of soluble sugars [50]. Also, a hampered build-up of non-structural carbohydrates in autumn due to defoliation can lead to a delayed bud burst in the next spring [51]. In our experiment, it could be postulated that the post-drought non-structural carbohydrate stores were possibly lowered both in the earlier senescing group of plants, because the growing season ended earlier, as in the later senescing group of plants, because resources were likely consumed in the repair of the hydraulic system, a process supported by the extended photosynthesis in later senescing leaves but not sufficient to bring the levels up to that of the control plants. Following this reasoning would imply that the treatment group with one- to three-quarters of desiccated leaves, due to the drought, which did not deviate from the controls in the timing of bud burst, succeeded in a build-up of non-structural carbohydrates up to the level of the controls. This hypothesis is supported by the recently observed strong relation between the levels of non-structural carbohydrates and the timing of bud burst in spring [52].

Population differentiation was present for the timing of bud burst in the common garden. The control plants of the Swedish provenance displayed both a later bud burst and an earlier leaf senescence when compared to the local provenance, indicating an adaptation to the shorter growing seasons at higher latitudes [53]. Control plants from the Spanish-Pyrenean provenance only displayed a later leaf senescence, but no differentiation in bud burst (lower latitude but also higher altitude). Whereas the population differentiation in the timing of leaf senescence disappeared for the plants that were more heavily affected by the drought, this phenomenon was not observed in the subsequent bud burst.

## 4. Materials and Methods

### 4.1. Study Species

*P. spinosa*, commonly known as blackthorn or sloe, is a deciduous thorny shrub with small, oval, serrated leaves and dark blue-black fruits called sloes. It blooms in early spring with white flowers before leaf emergence and is known for its dense growth, providing habitat and food for wildlife, including birds [54]. Fruits are rich in vitamin C and polyphenols, such as anthocyanins [55]. *P. spinosa* is a deciduous and widespread shrub species in Central and Southern Europe reaching up to Western Asia [54]. It is found in forest margins, wooded banks and hedgerows, prefers sunny and open spaces, and is adaptable to different soil conditions [54]. Hedgerows and wooded banks are valued not only for their role as barriers in the agricultural landscape but also for the wide range of ecosystem services they offer, including windbreaks and reduction of soil erosion [56]. In Belgium, it is often planted for species diversity, restoration of historical landscapes and to support wildlife [57]. *P. spinosa* is a widespread shrub species; however, it has received little attention in scientific research on woody species due to its lack of economic value. Still, some studies have emphasised key characteristics of the species regarding its morphology and genetics [58,59,60].

### 4.2. Plant Material

**Figure 8:**
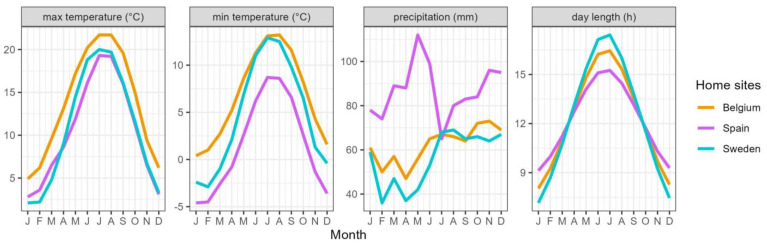
Mean monthly maximum and minimum temperature, mean monthly precipitation and day length at the origin of the provenances of *P. spinosa*.

We established a common garden of potted plants that consisted of 274 *P. spinosa* plants derived from three provenances: 107 plants from a local Belgian provenance (Lat 50.953324, Lon 3.663467 and Alt 10 m), 79 from a Spanish-Pyrenean provenance (Lat 42.630049, Lon −0.169068 and Alt 1270 m) and 88 from a south Swedish provenance (Lat 55.67668, Lon 13.32481 and Alt 58 m). Local climate and day length are shown in Figure 8 [53]. Stone collection has been described before [53]. In short, drupes were picked in 2016 and the stones germinated in 2017. From the beginning until the end of the period herein described, experiment plants were grown in pots using the same normal, commercially available nursery potting soil (1.5 kg/m^3^ NPK 12 + 14 + 24, 20% organic matter, pH levels from 5.0 to 6.5, electrical conductivity of 450 µS/cm, and 25% dry matter content), without addition of extra fertiliser. In 2018, a temperature experiment was conducted on the seedlings, and the effects of it were extinguished in 2020 [53]. A common garden of young plants in 1 L pots was created on an outdoor container field at the Research Institute for Agriculture and Fisheries (Melle, Belgium). The seedlings from the three provenances were intermingled in a single tree plot design. At the beginning of 2020, the plants were transferred to 4 L pots. The pots stayed on the container field throughout 2020 and into the beginning of 2021.

### 4.3. Drought Treatment

**Figure 9:**
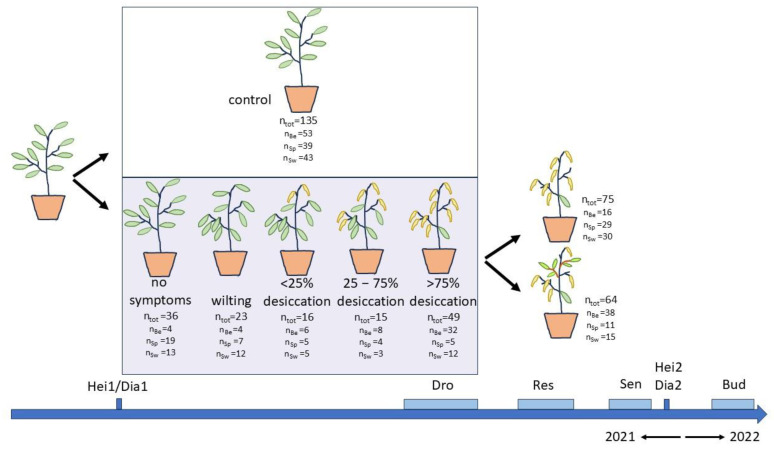
Schematic representation of the drought treatment with the plants subjected to water exclusion categorised according to the level of visible drought symptoms. The number of plants in each group is indicated with a further subdivision according to the provenance (Be: Belgian, Sp: Spanish-Pyrenean, Sw: Swedish). Several variables were recorded during and after the treatment. Hei1/Hei2/Dia1/Dia2: height and diameter, Dro: visual drought stress symptoms, Res: post-drought resprouting, Sen: autumn leaf senescence, Bud: bud burst.

In mid-June 2021, we moved all the potted plants from the container field to a greenhouse. We conducted a dry-out experiment from June 29 to July 29. The treatment started by placing all plants, including the controls, overnight in a water basin, as a proxy for field capacity. From now on, half of the plants received regular watering (control plants), while the other half received no water at all (droughted plants). The three provenances were evenly distributed between the control and drought groups (Figure 9) and were randomly intermingled in each group. To prevent excessive mortality, the drought treatment was stopped when several plants showed (nearly) total leaf desiccation. By this point, various visual drought stress symptoms were evident in the drought-treated plants (Figure 9). The treatment was ended by placing all plants, including the controls, overnight in a water basin.

After the treatment, the saplings were kept in a non-heated (but frost-free) greenhouse until January 2022, ensuring they remained consistently well watered. In January, the plants were planted in an experimental field in Grimminge, Belgium (single tree plot design).

### 4.4. Measurements and Observations

**Figure 10:**
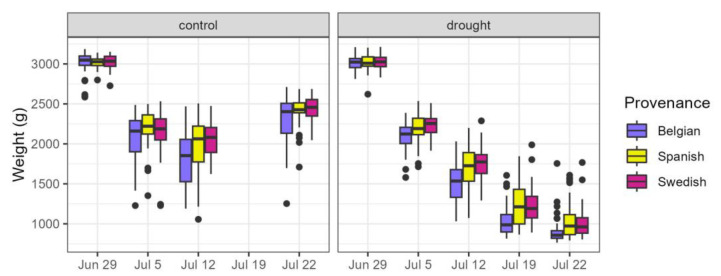
Weights of the pots during the treatment for the control and drought group, according to the provenance.

During and after the drought treatment, we performed various measurements and observations. All pots were weighed at the start of the treatment, after placing the pots overnight in a water basin and after draining excess water the next morning, and about weekly thereafter during the treatment (Figure 10). The decrease in pot weight during the drought period was an indicator of the water scarcity experienced by the drought-treated plants. The relative weight loss of the pots was calculated by subtracting the last weights from the initial weights at the beginning of the treatment and then dividing this difference by the initial weights.

Height and diameter were measured for all the plants at the start of the treatment (June 29), and when plants had entered winter rest (16 November). The height of the saplings was measured up to the place where the plants were still alive. The stem diameter was measured using a rod at 2 cm above the soil level.

We assessed the visual drought symptoms in the drought-treated group, i.e., the wilting and desiccation of the leaves, on 19, 22, 26 and 29 July, following a scoring protocol as follows: 1, no visual drought stress symptoms; 2, leaves wilting but not yet desiccating; 3, <25% of the leaves desiccated; 4, 25–75% of the leaves desiccated; and 5, >75% of the leaves desiccated (Figure 8). Five plants that lost (nearly) all their leaves due to desiccation by the drought did not resprout after the rewatering and thus were not recorded in the leaf senescence scoring, but they did flush in the next spring (four Belgian and one Swedish). Three plants finally died off, two of which lost more than 75% of their leaves due to the drought (both Belgian) and one with less than 25% desiccated leaves due to the drought (Swedish).

Plants in the drought-treated group were scored for resprouting after the rewatering, following a scoring protocol as follows: 1, buds not swelling; 2, buds swelling; 3, first leaves protruding but not yet unfolding; 4, first leaves unfolding; 5, first leaves unfolded but small; 6, first leaves enlarging; and 7, all new leaves on a plant enlarged. Resprouting was scored on 3, 10 and 17 August. After 17 August, no more plants started to resprout. A binary variable was deduced from the scorings on the last observation day (17 August), with 0 meaning plants not resprouting (score 1) and 1 meaning plants resprouting (scores > 1).

Autumn leaf senescence was scored applying the following protocol: 1, green leaves; 2, light green leaves; 3, less than half of the leaves turning yellow; 4, more than half of the leaves yellowing; 5, all leaves yellow and starting to fall off [53]. This phenophase was recorded on 21 September and 5 and 19 October. Bud burst in the spring of 2022 was evaluated applying the following protocol: 1, winter rest; 2, buds swell; 3, buds open and first leaves protrude but do not yet unfold; 4, leaves unfold; and 5, leaves unfolded and enlarged [53]. Bud burst was scored on 7, 14 and 21 April. For both phenophases, the whole sapling (i.e., all buds or all non-desiccated leaves) was assessed, and a mean score was assigned.

The relative chlorophyll content index in the leaves of a subset of plants was measured by making use of a chlorophyll content meter (CCM-200, Edaphic Scientific, Melbourne, Australia). The instrument determines the relative chlorophyll content of a leaf by calculating the ratio of optical transmission at 931 nm (near-infrared) to that at 653 nm (red). We focused on control plants at the one hand and plants that had reached severe visual drought symptoms during the drought period at the other hand. In the control group, 27 plants were chosen at random and in the group of drought-treated plants, 27 were chosen that displayed severe visual drought symptoms (3 with a visual drought symptoms score of 4 and 24 with a score of 5). In the middle of the crown, a representative mature leaf on a short shoot was carefully chosen for the measurement and marked. Repeated measurements were made on the same leaves, on 21 September, 5 October, 19 October and 2 November.

The leaf traits lamina length and widest width were measured on the first fully developed and damage-free leaf at the top of a representative long shoot at the top of the plant and on a fully developed leaf on a short shoot at the centre of the plant for all plants in the control group in the summer of 2021. For 57 randomly chosen plants (20 Belgian, 19 Spanish and 18 Swedish) in the control group, the stomatal density and stomatal length on the underside of the sampled leaves were counted and measured. A transparent nail varnish imprint was taken from the underside of the leaf at the centre of the leaf but avoided the veins. These imprints were placed on microscope slides and examined using a Keyence VHX-7000 digital microscope (Keyence Corporation, Osaka, Japan). In each nail varnish imprint, two stomatal counts, each in a randomly chosen square of 0.0454 mm^2^, were performed. The lengths of five randomly selected stomata in each square were measured.

### 4.5. Statistical Analysis

We used the open-source statistical software R, version 4.4.2 [61]. Linear models were applied to analyse height, diameter, leaf and stomatal size measurements. Logistic regression models were applied for the post-drought resprouting [62]. For phenological observations (the timing of visual drought symptoms, timing of resprouting, timing of leaf senescence, and timing of bud burst), which were ordinal data, we utilised cumulative logistic regression with the ordinal package [63]. Figures were created using ggplot2 [64]. Where applicable, a unique plant identifier was included as a random effect in the model to account for repeated observations of the same plants. Mixed-effects modelling is particularly well suited for analysing ecological data, as it can account for nested structures, handle unbalanced datasets, and incorporate random effects [65]. Variable abbreviations and descriptions are in Table 9.

Height (Hei1) and diameter (Dia1) at the start of the treatment were modelled to detect initial growth variations between the provenances.


Hei1 = β_0_ + β_1_Pro

Dia1 = β_0_ + β_1_Pro

The timing of the appearance of visual drought symptoms (Dro) in the drought-treated group of plants was modelled, with p_Dro_ being the chance to have reached maximally a given drought score level on a given day.


(p_Dro_/1 − p_Dro_) = β_0_ − β_1_Day − β_2_Pro − β_3_Rwe − β_4_Hei1

For the drought-treated plants, the chance to resprout after the rewatering (Res1) was modelled. For this, scorings of resprouting on the last observation day (17 August) were transformed to binary data (resprouting or not resprouting). The first model focussed on the influence of the visual drought symptoms.


(p_Res1_/1 − p_Res1_) = β_0_ + β_1_Dro + β_2_Hei1

When taking into account the provenance (Res1_Pro_), some levels of visual drought symptoms were pooled to attain a higher number of plants for each provenance in each droughted group. The dataset was split in two according to the two pooled levels of visual drought symptoms, to keep models as simple as possible.


(p_Res1Pro_/1 − p_Res1Pro_) = β_0_ + β_1_Pro + β_2_Hei1

For the droughted plants that recovered after rewatering by resprouting (n = 64, Figure 9), the timing of the resprouting (Res2) was modelled. Because of the low number of resprouting plants in the different score levels of visual drought symptoms, the lower drought score levels were pooled. p_Res2_ was the chance to have maximally reached a given resprouting score on a given day.


(p_Res2_/1 − p_Res2_)= β_0_ − β_1_Day − β_2_Dro_adj − β_3_Hei1

Because of the low number of plants for each provenance among the droughted plants that resprouted, the model for the timing of the resprouting (Res2_Pro_) containing the provenance in the fixed part, did not retain a visual drought symptoms variable.


(p_Res2Pro_/1 − p_Res2Pro_)= β_0_ − β_1_Day − β_2_Pro − β_3_Hei1

The timing of leaf senescence in autumn (Sen) was modelled for controls and drought treated plants together. Firstly, a model was run focussing on the different categories of visual drought symptoms. p_Sen_ was the chance to have maximally reached a given senescence score level on a given day.


(p_Sen_/1 − p_Sen_) = β_0_ − β_1_Day − β_2_Dro − β_3_Hei2

Secondly, the provenances were taken into account, and again, we had to take care of some drought categories with a limited number of plants for each provenance. Three datasets were constructed, and a model was run for each to keep the models as simple as possible. p_SenPro_ was the chance to have maximally reached a given senescence score level on a given day.


(p_SenPro_/1 − p_SenPro_) = β_0_ − β_1_Day − β_2_Pro − β_3_Hei2

For all the leaf senescence models (the visual drought symptoms model and the provenance models), time spans were calculated between the different groups of drought categories or between the provenances. The basic formula to calculate the day when 50% of the plants in a given group of plants had attained maximally a given leaf senescence score (Day_50%_) was based on (p_Sen_/1 − p_Sen_) being 0 for p_Sen_ = 0.5. Using the mean height at winter rest (mHei2):


Day_50%_ = (β_0_ − β_2_ − β_3_mHei2)/β_1_

Time spans for the timing of leaf senescence between two groups of plants (visual drought symptom groups or provenances) were calculated by subtracting the respective Day_50%_ values.

The relative chlorophyll content measurements of the leaves (Rcc) were analysed. Because there were too few measurements for every provenance separately, provenance was not taken into account in the model. Because the measurements were not linear over time, the time variable was quadratic in the model. An interaction term between the time variable and the adjusted visual drought symptoms variable (Dro_adj2) allowed the relative chlorophyll content to diminish over time at a different rate for the different levels in the Dro_adj2 variable.


Rcc = β_0_ + β_1_Day + β_2_ Day^2^ + β_3_Dro_adj2 + β_4_Day:Dro_adj2 + β_5_Day^2^:Dro_adj2

In the year after the drought treatment, bud burst (Bud) was scored on all the plants. We modelled the probability (p_Bud_) that on a given day, a sapling had already reached a given bud burst score or a score higher than this. First, we focussed on the different visual drought symptom categories.


(p_Bud_/1 − p_Bud_) = β_0_ − β_1_Day − β_2_Dro − β_3_Hei2

Similar to the timing of leaf senescence, the timing of bud burst in the three provenances (p_BudPro_) was studied in three datasets.


(p_BudPro_/1 − p_BudPro_) = β_0_ − β_1_Day − β_2_Pro − β_3_Hei2

Leaf lamina length (Lle) and lamina widest width (Llw) were analysed using a linear model.


Lle = β_0_ + β_1_Pro

Llw = β_0_ + β_1_Pro

For stomatal length (Stl), a linear mixed model was applied.


Stl = β_0_ + β_1_Pro

Finally, for the stomatal density (Std), a Poisson general linear mixed model was applied.


Std = β_0_ + β_1_Pro

## 5. Conclusions

With climate change, extreme droughts are expected to become more frequent. Our study showed that *P. spinosa* can resprout even after a severe summer drought, indicating that this common shrub species evolved rescue mechanisms to enhance survival, albeit at the cost of a higher mortality risk if subsequent droughts would occur. Surprisingly, leaf senescence was advanced or delayed depending on the severity of the drought stress. While earlier senescence reduces photosynthetic activity, delayed senescence serves as a recovery strategy that also implies a higher risk of damage by early autumn frosts.

Population differentiation was observed in the drought responses. The local provenance exhibited stress symptoms more quickly during the drought but also had a higher likelihood of post-drought resprouting. The differentiation in timing of leaf senescence between the provenances faded among the more severely affected plants by the drought, exemplifying how changes in the growth environment of plants can shape their responses in such a way that their genetic patterns become less clear. Our results remain inconclusive on whether local populations could be more or less resilient to increasing drought intensity under climate change.

## Figures and Tables

**Table 1:** Test statistics for the height (Hei1) and diameter (Dia1) at the start of the treatment, with *p* values for the fixed effects in the mixed model. The Spanish-Pyrenean (Sp) and the Swedish (Sw) provenances are compared to the standard Belgian provenance.

	Height				Diameter			
	Estimate	Std. Error	t Value	*p* Value	Estimate	Std. Error	t Value	*p* Value
(Intercept)	75.523	1.055	71.557	<0.001 ***	8.900	0.113	78.898	<0.001 ***
Sp	−10.650	1.619	−6.576	<0.001 ***	−0.617	0.173	−3.564	<0.001 ***
Sw	−5.194	1.571	−3.306	0.001 **	−1.214	0.168	−7.231	<0.001 ***

*** *p* < 0.001; ** *p* < 0.01.

**Table 2:** Test statistics for the development of drought symptoms during the drought treatment. The Spanish-Pyrenean (Sp) and the Swedish (Sw) provenances are compared to the standard Belgian provenance. Rew is the relative weight loss, Day is the day of observation, and Hei1 is the plant height at the start of the treatment.

	Estimate	Std. Error	z Value	*p* Value
Day	0.707	0.057	12.330	<0.001 ***
Sp	−3.416	0.815	−4.194	<0.001 ***
Sw	−1.754	0.713	−2.459	0.014 *
Rew	77.327	9.396	8.230	<0.001 ***
Hei1	0.053	0.028	1.930	0.054

*** *p* < 0.001; * *p* < 0.05.

**Table 3:** Test statistics for the modelling of the chance on post-drought resprouting in the drought-treated plants among the different categories of visual drought symptoms (Dro) and among the provenances, with *p* values for the fixed effects in the mixed model. In the model of the drought categories, the category normal (no symptoms, score 1) is the standard to which the other categories are compared to. In the two provenance models, the Belgian provenance is the standard to which the other provenances are compared to. Hei1 is the plant height at the start of the drought period. Sp: Spanish-Pyrenean provenance, Sw: Swedish provenance.

		Estimate	Std. Error	z Value	*p* Value
Visual drought symptom categories (Dro)	(Intercept)	−2.477	1.703	−1.454	0.146
	Dro score 2	−0.238	1.258	−0.190	0.850
	Dro score 3	2.341	0.897	2.612	0.009 **
	Dro score 4	4.766	1.087	4.383	<0.001 ***
	Dro score 5	4.692	0.888	5.284	<0.001 ***
	Hei1	−0.006	0.024	−0.231	0.818
provenances in dataset containing no to mild visual drought symptom categories (pooled Dro scores 1, 2 and 3)	(Intercept)	1.511	2.561	0.590	0.555
	Sp	−2.320	0.972	−2.387	0.017 *
	Sw	−2.136	0.933	−2.289	0.022 *
	Hei1	−0.031	0.037	−0.831	0.406
provenances in dataset containing severe visual drought symptoms (pooled Dro scores 4 and 5)	(Intercept)	0.625	2.578	0.242	0.808
	Sp	17.060	2171.000	0.008	0.994
	Sw	0.310	0.868	0.358	0.721
	Hei1	0.012	0.034	0.362	0.718

*** *p* < 0.001; ** *p* < 0.01; * *p* < 0.05.

**Table 4:** Test statistics for the modelling of the timing of post-drought resprouting in the resprouting plants of the drought-treated group, among the different categories of visual drought symptoms and among the provenances, with *p* values for the fixed effects in the mixed model. In the model of the drought categories, the categories normal, wilting and <25% desiccated leaves (Dro scores 1, 2 and 3) are pooled and serve as the standard to which the other categories are compared to. In the provenance model, the Belgian provenance is the standard to which the other provenances are compared to. Day is the day of observation, and Hei1 is the plant height at the start of the treatment. Sp: Spanish-Pyrenean provenance, Sw: Swedish provenance.

		Estimate	Std. Error	z Value	*p* Value
visual drought symptom categories (Dro with scores 1, 2 and 3 being pooled)	Day	−0.885	0.098	−9.011	<0.001 ***
	Dro score 4	−2.774	1.258	−2.206	0.027 *
	Dro score 5	−4.498	1.149	−3.916	<0.001 ***
	Hei1	−0.029	0.030	−0.986	0.324
provenances	Day	−0.883	0.097	−9.073	<0.001 ***
	Sp	0.879	1.072	0.820	0.412
	Sw	0.344	0.955	0.360	0.719
	Hei1	−0.060	0.034	−1.775	0.076

*** *p* < 0.001; * *p* < 0.05.

**Table 5:** Test statistics for the modelling of the timing of leaf senescence in controls and droughted plants, among the different categories of visual drought symptoms and among the provenances, with *p* values for the fixed effects in the mixed model. In the visual drought symptoms model, the control plants are the standard to which the different categories of visual drought symptoms (Dro) are compared to. In the three provenance models, the Belgian provenance is the standard to which the other provenances are compared to. Day is the day of observation, and Hei2 is the plant height at the end of the growing season. Sp: Spanish-Pyrenean provenance, Sw: Swedish provenance.

		Estimate	Std. Error	z Value	*p* Value
visual drought symptom categories (total dataset)	Day	0.343	0.020	17.288	<0.001 ***
	Dro score1	−0.647	0.347	−1.864	0.062
	Dro score2	0.941	0.398	2.363	0.018 *
	Dro score3	−0.084	0.464	−0.180	0.857
	Dro score4	−2.379	0.524	−4.545	<0.00 1***
	Dro score5	−5.126	0.442	−11.609	<0.001 ***
	Hei2	0.013	0.009	1.516	0.129
provenances in dataset of control plants	Sp	−0.872	0.311	−2.808	0.005 **
	Sw	0.808	0.272	2.969	0.003 **
	Day	0.334	0.026	12.699	<0.001***
	Hei2	0.008	0.008	0.968	0.333
provenances in dataset of plants with no to mild visual drought symptoms (pooled Dro scores 1, 2 and 3)	Sp	−1.771	0.673	−2.631	0.009 **
	Sw	1.239	0.634	1.954	0.051
	Day	0.379	0.041	9.248	<0.001 ***
	Hei2	0.003	0.021	0.134	0.893
provenances in dataset of plants with severe visual drought symptoms (pooled Dro scores 4 and 5)	Sp	−0.694	1.052	−0.659	0.510
	Sw	0.569	0.872	0.652	0.514
	Day	0.390	0.064	6.129	<0.001 ***
	Hei2	−0.006	0.033	−0.172	0.863

*** *p* < 0.001; ** *p* < 0.01; * *p* < 0.05.

**Table 6:** Test statistics for the modelling of the relative chlorophyll content index between control plants and plants that lost more than a quarter of their foliage due to the drought, with *p* values for the fixed effects in the mixed model. The control plants are the standard to which the severely affected plants (Dro_adj2) are compared to. Day is the day of observation.

	Estimate	Std. Error	DF	t Value	*p* Value
(Intercept)	11.45	0.69	152	16.65	<0.001 ***
Day	−55.78	3.21	152	−17.37	<0.001 ***
Day^2^	−18.04	3.15	152	−5.72	<0.001 ***
Dro_adj2	1.98	0.97	52	2.04	0.047 *
Day:Dro_adj2	15.49	4.40	152	3.52	0.001 ***
Day^2^:Dro_adj2	4.00	4.38	152	0.91	0.362

*** *p* < 0.001; * *p* < 0.05.

**Table 7:** Test statistics for the modelling of the timing of bud burst in controls and droughted plants, among the different categories of visual drought symptoms and among the provenances, with *p* values for the fixed effects in the mixed model. In the visual drought symptoms model, the control plants are the standard to which the different categories of visual drought symptoms (Dro) are compared to. In the three provenance models, the Belgian provenance is the standard to which the other provenances are compared to. Day is the day of observation, Hei2, the plant height at the end of the growing season. Sp: Spanish-Pyrenean provenance, Sw: Swedish provenance.

		Estimate	Std. Error	z Value	*p* Value
visual drought symptom categories (total dataset)	Dro score1	0.450	0.621	0.724	0.469
	Dro score2	2.374	0.756	3.139	0.002 **
	Dro score3	1.839	0.902	2.038	0.042 *
	Dro score4	0.366	0.895	0.409	0.682
	Dro score5	1.901	0.565	3.363	<0.001 ***
	Hei2	0.058	0.014	4.028	<0.001 ***
	Day	−0.914	0.051	−17.785	<0.001 ***
provenances in dataset of control plants	Sp	−0.713	0.507	−1.407	0.160
	Sw	4.286	0.572	7.490	<0.001 ***
	Day	−0.854	0.069	−12.427	<0.001 ***
	Hei2	0.061	0.015	4.033	<0.001 ***
provenances in dataset of plants with no to mild visual drought symptoms (pooled Dro scores 1, 2 and 3)	Sp	0.350	0.750	0.466	0.641
	Sw	4.977	0.920	5.410	<0.001 ***
	Day	−0.889	0.099	−9.017	<0.001 ***
	Hei2	0.105	0.027	3.913	<0.001 ***
provenances in dataset of plants with severe visual drought symptoms (pooled Dro scores 4 and 5)	Sp	−0.114	1.198	−0.095	0.924
	Sw	4.829	1.170	4.129	<0.001 ***
	Day	−1.036	0.131	−7.894	<0.001 ***
	Hei2	−0.004	0.037	−0.111	0.912

*** *p* < 0.001; ** *p* < 0.01; * *p* < 0.05.

**Table 8:** Schematic representation of the timing of autumn leaf senescence and spring bud burst in the droughted plants in comparison to the controls. Droughted plants are grouped according to the categories of visual drought symptoms during the summer water withholding.

Visual Drought Symptoms During Water Withholding (Dro)	Leaf Senescence	Bud Burst
Score 1: no symptoms	=control	=control
Score 2: wilting leaves	earlier	later
Score 3: <25% desiccated leaves	=control	later
Score 4: 25–75% desiccated leaves	later	=control
Score 5: >75% desiccated leaves	later	later

**Table 9:** Description of the variables in the statistical modelling.

Abbreviation	General Description	Detailed Description
Bud	Timing of bud burst: model without provenance	
Bud_Pro_	Timing of bud burst: model with provenance	Three datasets: Control plants + Pooled Dro scores 1, 2 and 3 + pooled Dro scores 4 and 5
Day	Day of observation	
Dia1	Initial diameter	
Dro	Visual drought symptoms	
Dro_adj	Pooled visual drought symptoms	Categorical variable with 3 levels: Pooled Dro scores 1, 2 and 3 + Dro score 4 + Dro score 5
Dro_adj2	Controls and pooled visual drought symptoms	Categorical variable with 2 levels: Controls + pooled Dro scores 4 and 5
Hei1	Initial height	
Hei2	Height at winter rest	
Lle	Leaf lamina length	
Llw	Leaf lamina widest width	
Pro	Provenance	Categorical variable with 3 levels: Be (Belgian) + Sp (Spanish-Pyrenean) + Sw (Swedish)
Rcc	Relative chlorophyll content	
Res1	Resprouting (yes/no): model without provenance	
Res1_Pro_	Resprouting (yes/no): model with provenance	Two datasets: Pooled Dro scores 1, 2 and 3 + pooled Dro scores 4 and 5
Res2	Timing of resprouting: model without provenance	
Res2_Pro_	Timing of resprouting: model with provenance	
Rwe	Relative weight loss of the pots	
Sen	Timing of leaf senescence: model without provenance	
Sen_Pro_	Timing of leaf senescence: model with provenance	Three datasets: Control plants + Pooled Dro scores 1, 2 and 3 + pooled Dro scores 4 and 5
Stl	Stomatal length	
Std	Stomatal density	

## Data Availability

Data are available at zenodo 10.5281/zenodo.13838214 (https://zenodo.org/search?q=13838214&l=list&p=1&s=10&sort=bestmatch) (community: genfored).

## Supplementary Materials

The following supporting information can be downloaded at: https://www.mdpi.com/article/10.3390/plants14071132/s1, Figure S1: Boxplots displaying the lamina length and lamina widest width of representative mature leaves on long shoots and short shoots of the control plants, according to their provenance. Be: Belgian, Sp: Spanish-Pyrenean, Sw: Swedish; Figure S2: Stomatal density (a) and stomatal length (b) of representative and mature long shoot leaves in the three provenances. Be: Belgian, Sp: Spanish-Pyrenean, Sw: Swedish; Figure S3: Modelled probability of the timing of bud burst for controls and for droughted plants (according to visual drought symptom categories), depending on the provenances. Provenances not significantly differing from the standard (Belgian) are displayed in grey; Table S1: Test statistics for the length and widest width of the lamina of mature leaves on long and short shoots of the control plants. The Belgian provenance is the standard to which the Spanish-Pyrenean (Sp) and the Swedish (Sw) provenances are compared to; Table S2: Test statistics for the density and the length of stomata of mature leaves on long shoots of the control plants. The Belgian provenance is the standard to which the Spanish-Pyrenean (Sp) and the Swedish (Sw) provenances are compared to.
