# Supplementary material for: Influence of Summer Drought on Post-Drought Resprouting and Leaf Senescence in Prunus spinosa L. Growing in a Common Garden
Source: Plants (Basel). 2025 Apr 5;14(7):1132. doi: 10.3390/plants14071132 (PMC11991280; doi:10.3390/plants14071132)
Supplement: Supplementary file 1 [file plants-14-01132-s001.zip › plants-3500673-supplementary.pdf]

Supplementary Materials

Type of the Paper: Article

Influence of Summer Drought on Post-drought Resprouting and Leaf Senescence in *Prunus spinosa* L. growing in a Common Garden.

Kristine Vander Mijnsbrugge [1]*,Stefaan Moreels [1],Sharon Moreels [1],Damien Buisset[1], Karen Vancampenhout[2] and Eduardo Notivol Paino[3]

[1] Department of Forest Ecology and Management, Research Institute for Nature and Forest, 9500 Geraardsbergen, Belgium; stefaan.moreels@inbo.be; Sharon.moreels@inbo.be; damien.buisset@ulb.be
[2] Department of Earth and Environmental Sciences, KU Leuven Campus Geel, Kleinhoefstraat 4, 2440 Geel, Belgium; karen.vancampenhout@kuleuven.be
[3] Department for Environment, Agricultural and Forest Systems, Agri-Food Research and Technology Centre of Aragon (CITA), Spain; enotivol@cita-aragon.es
* Correspondence: kristine.vandermijnsbrugge@inbo.be

1. Figures

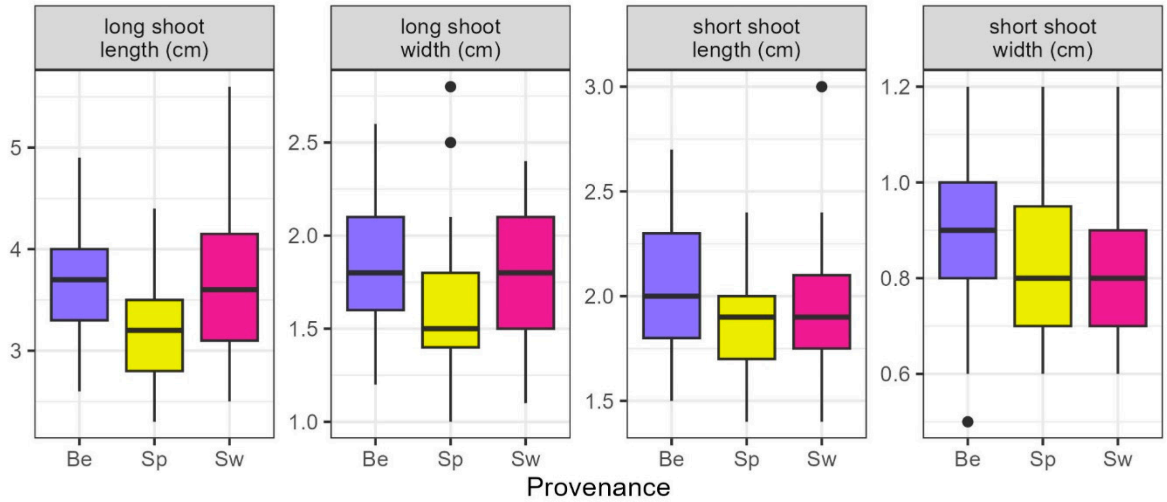

Figure S1. Boxplots displaying the lamina length and lamina widest width of representative mature leaves on long shoots and short shoots of the control plants, according to their provenance. Be: Belgian, Sp: Spanish-Pyrenean, Sw: Swedish.

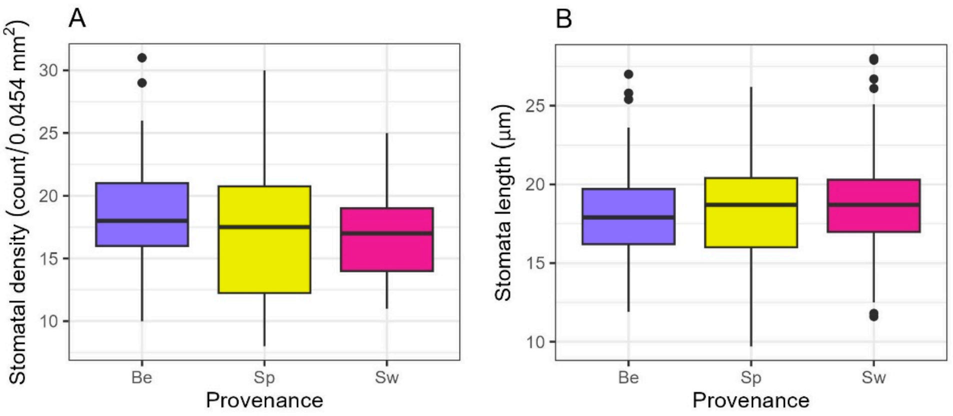

(**a**) (**b**)

Figure S2. Stomtal density (**a**) and stomatal length (**b**) of representative and mature long shoot leaves in the three provenances. Be: Belgian, Sp: Spanish-Pyrenean, Sw: Swedish.

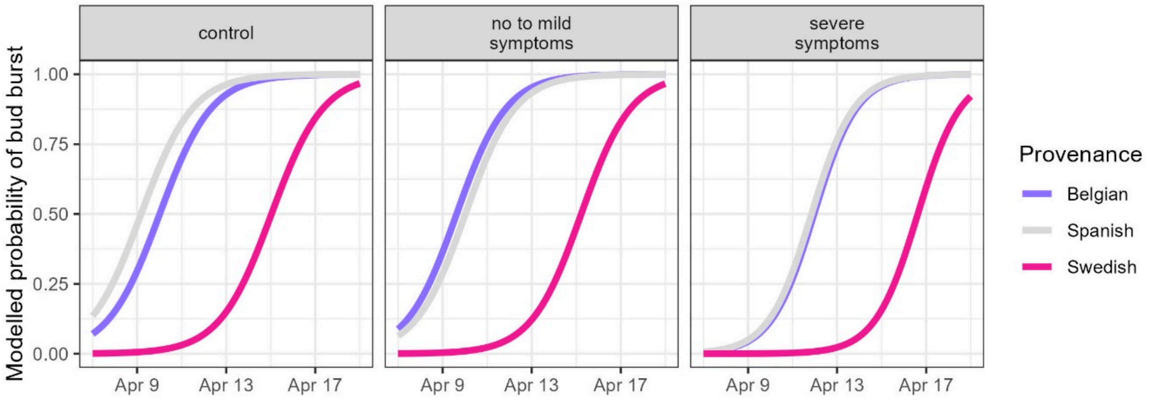

Figure S3. Modelled probability of the timing of bud burst (having already leaves emerging from the bud) for controls and for droughted plants (according to visual drought symptom categories), depending on the provenances. Provenances not significantly differing from the standard (Belgian) are displayed in grey.

2. Tables

Table S1. Test statistics for the length and widest width of the lamina of mature leaves on long and short shoots of the control plants, with p values for the independent variables in the models. The Belgian provenance is the standard to which the Spanish-Pyrenean (Sp) and the Swedish (Sw) provenances are compared to.

Response variable		long shoot				short shoot			
		Estimate	Std. error	t value	p value	Estimate	Std. error	t value	p value
lamina length	(Intercept)	3,662	0,087	42,221	<0,001***	2,028	0,041	49,496	<0,001
	Sp	-0,503	0,133	-3,778	<0,001***	-0,164	0,063	-2,609	0,010*

	Sw	0,103	0,130	0,794	0,429	-0,107	0,061	-1,754	0,082
lamina	(Intercept)	1,866	0,050	37,658	<0,001***	0,883	0,021	41,624	<0,001
widest	Sp	-0,235	0,076	-3,091	0,002**	-0,032	0,033	-0,974	0,332
width	Sw	-0,075	0,074	-1,018	0,311	-0,074	0,032	-2,326	0,022*

Table S2. Test statistics for the density and the length of stomata of mature leaves on long shoots of the control plants, with p values for the fixed effects in the mixed models. The Belgian provenance is the standard to which the Spanish-Pyrenean (Sp) and the Swedish (Sw) provenances are compared to.

	stomatal density				stomata length				
	Estimate	Std. error	z value	p value	Estimate	Std. error	DF	t value	p value
(Intercept)	2,898	0,054	53,877	<0,001***	18,060	0,436	532	41,375	<0,001***
Sp	-0,083	0,078	-1,054	0,292	0,164	0,634	55	0,259	0,797
Sw	-0,087	0,079	-1,103	0,270	0,679	0,617	55	1,099	0,2765
